# An Evaluation of the Accuracy of a Flash Glucose Monitoring System in Children with Diabetes in comparison with Venous Blood Glucose

**DOI:** 10.1155/2019/4845729

**Published:** 2019-09-09

**Authors:** Bingyan Cao, Rui Wang, Chunxiu Gong, Di Wu, Chang Su, Jiajia Chen, Yajun Yi, Min Liu, Xuejun Liang, Wenjing Li

**Affiliations:** Department of Endocrinology, Genetic and Metabolism, Beijing Children's Hospital, Capital Medical University, National Center for Children's Health, Beijing 100045, China

## Abstract

**Aims:**

To evaluate the performance of a factory-calibrated flash glucose monitoring system in children with diabetes compared to venous blood glucose (BG).

**Methods:**

A total of 13 hospitalized participants newly diagnosed with type 1 diabetes, aged 1~14 years old, were involved in the study. Sensor glucose measurements on days 2, 3, 6, 7, 12, and 13 of wear were compared with venous BG. During these days, the venous BG results were obtained either 4 or 7 times per day.

**Results:**

The accuracy was evaluated against venous BG, with 469 of 469 (100.0%) sensor and venous BG pairs within consensus error grid zones A and B, including 94.7% in zone A. The overall mean absolute relative difference (MARD) was 11.67%. The MARD of blood glucose lower than 4.0 mmol/L (MARD = 16.89%) was higher than blood glucose between 4 and 10 mmol/L (MARD = 11.58%) and blood glucose higher than 10 mmol/L (MARD = 7.79%). Compared to venous BG, the MARDs of wear days 2, 3, 6, 7, 12, and 13 were 11.53%, 9.66%, 11.79%, 10.89%, 13.18%, and 13.92%, respectively, with no statistically significant difference (*P* = 0.25). The median ARD was highest when the glucose decreased >0.11 mmol/L/min (20.27%) and lower than 10.00% when the glucose changed between 0.06 and 0.11 mmol/L/min, changed <0.06 mmol/L/min, and increased >0.11 mmol/L/min.

**Conclusions:**

The accuracy of the system is good and remains stable over 14 days of wear; however, the accuracy depends on the glucose level and rates of glucose concentration changes.

## 1. Introduction

Continuous glucose monitoring (CGM) can provide continuous real-time measurement of glucose levels, alerts for hyperglycaemia and hypoglycaemia, and assessment of glucose variation. CGM can also help improve the glucose control in type 1 diabetics, so the international guidance supports the use of CGM for people with type 1 diabetes [1]. Moreover, CGM is safe and efficacious for the paediatric population based on randomized controlled trials on CGM use [2]. The challenge to date was the expense and extra calibration of capillary blood glucose.

The FreeStyle Libre™ Flash glucose monitoring system (Abbott Diabetes Care, Alameda, CA) is an interstitial glucose monitoring system, which has several features that distinguish it from the existing sensor technology. First, this system measures interstitial glucose levels for up to 14 days without calibration by fingerstick blood glucose measurements. Second, the wireless handheld reader scans the sensor every 15 minutes for up to 8 h to receive the glucose result and trend. Several studies have demonstrated the accuracy of this system by comparing the sensor results with capillary BG values and venous YSI measurements in adults [3, 4]. The accuracy has been evaluated in children in several studies through comparison of the sensor results with capillary BG values [5–7], in trial or real-life settings, and also analysis of the factors that might affect its accuracy, including stable or fluctuating glycaemic conditions and time of day. The results have demonstrated that the accuracy depends on the glucose trend [6]; however, all the reported data in paediatric populations used capillary BG as a reference, which is affected by many factors and does not reflect the real blood glucose level. Venous BG should also be compared when evaluating the accuracy and performance of the CGM [8–11]. There were no data on comparison of the sensor results and venous values. Furthermore, the China Food and Drug Administration (CFDA) has not approved this system for use in Chinese children and adolescents. We first evaluated the performance and usability of a factory-calibrated flash glucose monitoring system against venous BG in a Chinese paediatric diabetes population.

## 2. Materials and Methods

This study was authorized by the Ethics Committee of Beijing Children's Hospital affiliated with the Capital Medical University (Beijing, China). All parents of the children enrolled in this study were informed and provided signed consent to provide the data for this study.

A total of 13 hospitalized children with newly diagnosed type 1 diabetes were enrolled in the study. The participants wore the sensor on the back of the upper arm without any over-bandage for up to 14 days. Sensor glucose measurements on days 2, 3, 6, 7, 12, and 13 were compared with venous BG. During these days, the venous BG results were obtained either 4 or 7 times per day (<4 years old, 4 times per day, and >4 years old, 7 times per day). Sensor scans were performed each time a venous blood sample was taken. The venous BG was measured using the glucose oxidase method within 15 minutes by an automatic chemistry analyser (Beckman Coulter AU5800, America Ltd.). The preferred testing was upon waking, before each meal, 2 hours after each meal, and at bedtime. For the single patient younger than 4 years old, 4 venous blood samples were taken per day which included at waking, before lunch and supper, and 2 hours after supper. Three factory-calibrated production sensor lots were used in this study. This number is consistent with industry practice to demonstrate the performance of reagent systems across multiple production lots.

The consensus error grid (CEG) and Clarke error grid were carried out to evaluate the performance and efficacy for making clinical decisions. This method allows one to identify the frequency of errors and the device performance according to the zones A, B, C, D, and E. The higher the percentages in zone A and zones A+B, the better device performance.

The concordance correlation coefficient (CCC) index measures the correlation and concordance between the sensor glucose and venous BG. We also performed a regression analysis of the sensor glucose compared with venous BG on different days of sensor wear and at different blood glucose levels.

We use a number of methods to evaluate the accuracy of the system including the mean absolute difference (MAD) that measures the size of difference and the mean absolute relative difference (MARD) that measures the size of the differences compared with the reference as a percentage of the reference value to evaluate the accuracy at low and mid to high glucose ranges and on different days of sensor wear. We analysed the difference between the sensor glucose and venous BG and median ARD on different rates of glucose concentration changes. The rates of change in the glucose concentration are defined by the FreeStyle Libre arrows as follows: ↑ means increasing >0.11 mmol/L/min, ↗ means increasing between 0.06 and 0.11 mmol/L/min, → means changing <0.06 mmol/L/min, ↘ means decreasing between 0.06 and 0.11 mmol/L/min, and ↓ means decreasing >0.11 mmol/L/min.

All the data were analysed using SAS JMP version 11 (SAS Institute Inc., Cary, NC) and MedCalc Statistical Software version 12.7.8 (MedCalc Software bvba, Ostend, Belgium; http://www.medcalc.org; 2014).

## 3. Results

The mean age of the participants was 8.69 ± 4.1 years and included 5 males and 8 females. Among them, 53.8% used regular insulin injection and 47.2% used CSII. The mean HbA1c was 11.62 ± 2.85% (103.50 ± 7.65 mmol/mol). The baseline characteristics of the participants are shown in Table 1. Two participants only completed 10 days of sensor wear because of discharge, and the sensor of one child fell off on day 11 due to adhesive loss. Sixteen real-time sensor glucose readings were excluded because the venous blood measurements were delayed, and one pair was excluded because the sensor result was beyond the system's dynamic range (40-500 mg/dL). In total, we obtained 469 venous BG measurements paired with sensor glucose results.

**Figure 1:**
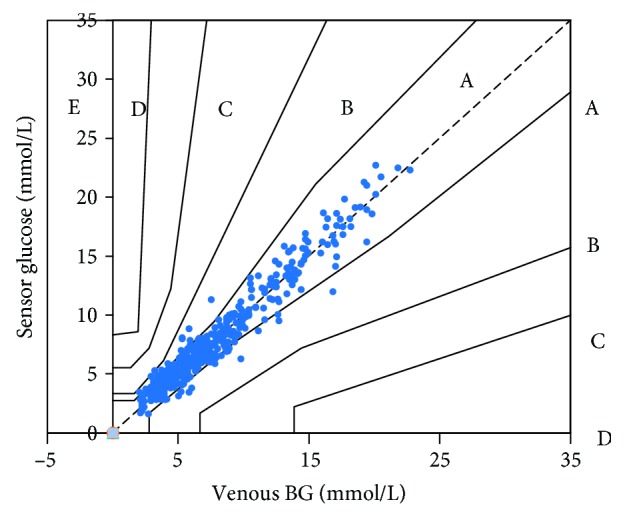
Consensus error grid (CEG) analysis comparing FreeStyle Libre sensor and venous blood glucose (BG) results.

The overall percentage of the results in zones A and B of the consensus and Clarke error grids was 100.0%, including 94.7% in zone A (Figure 1). The percentage of results within CEG zone A was similar for the subgroups (different days of wear, different glucose levels, and time of day) as shown in Table 2.

**Figure 2:**
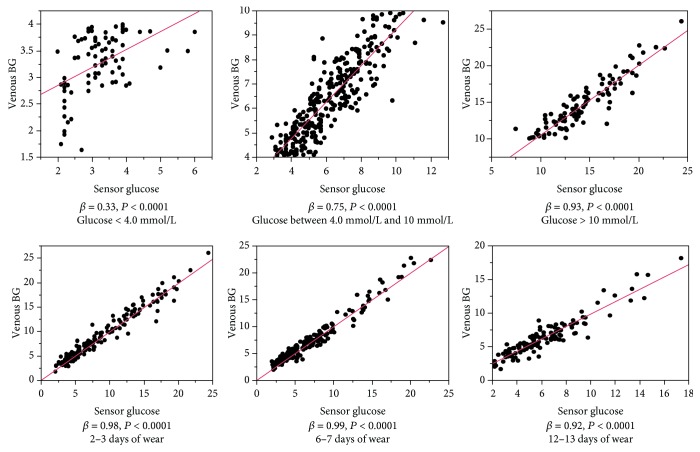
The regression analysis of sensor glucose compared with venous blood glucose on different days of sensor wear and different blood glucose levels.

The concordance correlation coefficient (CCC) of the sensor glucose compared with venous BG on different days of sensor wear and different blood glucose levels is shown in Table 3. With an overall CCC of 0.97, the agreement was best when the BG was higher than 10 mmol/L, followed by glucose levels between 4 and 10 mmol/L, and worst when the BG was lower than 4 mmol/L. The results of the regression analysis of the sensor glucose compared with the venous BG on different days of sensor wear and different blood glucose levels are shown in Figure 2.

**Figure 3:**
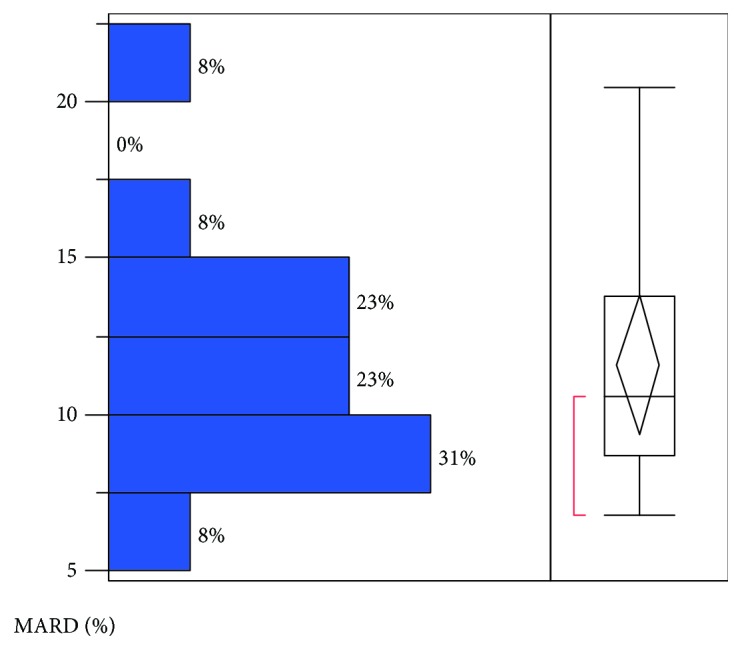
The variation of mean absolute relative difference (MARD) against venous BG for the different sensors.

The overall MARD was 11.67% for the sensor results with the venous BG reference. The variation in MARD against venous BG for the different sensors is shown in Figure 3. The MARD when the blood glucose was lower than 4.0 mmol/L (MARD = 16.89%) was higher than when the blood glucose was between 4 and 10 mmol/L (MARD = 11.58%) and higher than 10 mmol/L (MARD = 7.79%). Compared to venous BG, the MARDs of days 2, 3, 6, 7, 12, and 13 were 11.53%, 9.66%, 11.79%, 10.89%, 13.18%, and 13.92%, respectively, with no statistically significant difference (*P* = 0.25); therefore, the accuracy of the sensor was stable across the 2-week evaluation period. A detailed difference analysis against the venous BG reference is shown in Table 3. The MARD was higher during the nighttime (21:00~7:00) than in the daytime (7:00~21:00) (12.77% and 10.88%, respectively; *P* = 0.03).

**Figure 4:**
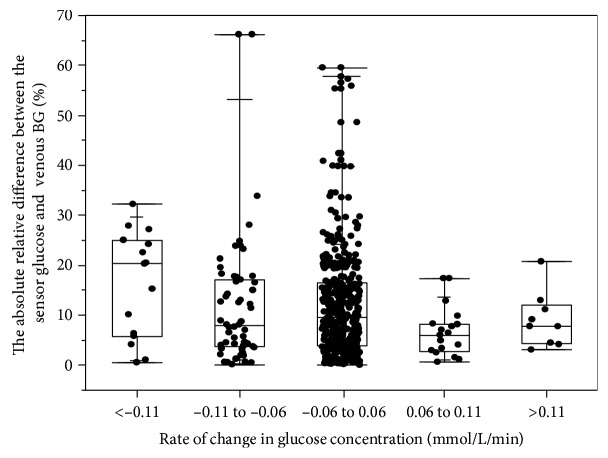
The absolute relative difference between the sensor glucose and venous BG results at different rates of glucose concentration changes.

The absolute relative differences (ARDs) of the sensor results compared to the venous BG at different rates of glucose concentration changes are shown in Figure 4. The median ARD was highest when the glucose decreased >0.11 mmol/L/min (20.27%) and lower than 10.00% when the glucose changed between 0.06 and 0.11 mmol/L/min, changed <0.06 mmol/L/min, and increased >0.11 mmol/L/min.

**Figure 5:**
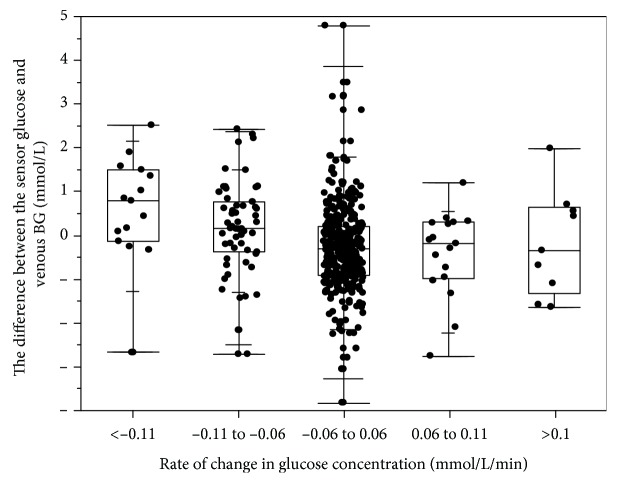
The difference between the sensor glucose and venous BG results (sensor glucose minus venous glucose) at different rates of glucose concentration changes.

In some sensor systems, sensor results are reportedly higher than the BG results when the glucose is decreasing and lower than the BG results when the glucose is increasing due to the lag between the sensor glucose and venous BG measurements [12]. We also found that the sensor glucose was higher than the venous BG when the glucose was decreasing and lower than the venous BG when the glucose was increasing, demonstrating the existence of a “lag” effect (Figure 5). The difference between the sensor glucose and the venous BG was the largest when the glucose decreased >0.11 mmol/L/min. The median absolute difference between the sensor glucose and the venous BG when glucose decreased >0.11 mmol/L/min, decreased between 0.06 and 0.11 mmol/L/min, changed <0.06 mmol/L/min, increased between 0.06 and 0.11 mmol/L/min, and increased >0.11 mmol/L/min was 0.79 mmol/L, 0.16 mmol/L, 0.30 mmol/L, 0.18 mmol/L, and 0.34 mmol/L, respectively. The MADs were 0.54 mmol/L and 0.47 mmol/L when BG <4.0 mmol/L and <5.55 mmol/L, respectively.

No swelling or skin redness during the 14 days of use was noted, and only one sensor detached because of adhesive loss.

## 4. Discussion

This study evaluated the performance and usability of a factory-calibrated flash glucose monitoring system in a paediatric diabetes population. This first study compared sensor glucose with venous BG. The system was found to be accurate with 100.0% sensor and venous BG pairs within consensus error grid zones A and B, including 94.7% in zone A, results that agree very closely with other similar studies in adults [3, 4]. The report from Edge et al. compared the sensor glucose measurements with capillary BG measurements in type 1 diabetic children aged 4~17 years [5]. The CEG analysis demonstrated 83.8% of results in zone A and 99.4% of results in zones A and B; however, the authors did not compare the sensor glucose with the venous BG levels. The overall MARD versus the venous BG of 11.67% in our study was similar as in adults (12.2%) [3]. The average MARD versus capillary BG levels were reported to be 13.5% and 13.9% in children [5, 6]. The overall CCC of the sensor glucose compared with venous BG was 0.97; therefore, our results further confirmed the accuracy of this system by comparing sensor glucose with venous BG.

The study also evaluated the accuracy of this system after different days of wear and at various glucose levels. There was no difference in the MARD on different days of wear. The MARD results were 11.53%, 9.66%, 11.79%, 10.89%, 13.18%, and 13.92% on days 2, 3, 6, 7, 12, and 13, respectively. The concordance correlation analysis showed that the sensor glucose and venous BG correlated very well during the 14 days of wear. The agreement slightly declined on days 12 and 13 of wear compared with days 2, 3, 6, and 7 of wear. The results demonstrate that the system was accurate over the 14-day period of sensor wear. Glucose levels affect the agreement between the venous BG and sensor glucose. The agreement was best when the glucose was higher than 10 mmol/L, followed by glucose levels between 4 and 10 mmol/L, and worst when the glucose was lower than 4 mmol/L. Additionally, the MARD when the blood glucose was lower than 4.0 mmol/L was 16.89%, much higher than when the blood glucose was between 4 and 10 mmol/L and higher than 10 mmol/L (11.58% and 7.79%, respectively). We therefore advise patients to use a BG meter to check the sensor result if the result is lower than 4.0 mmol/L or the symptoms do not match the sensor result.

The rates of change in the glucose concentration affected the accuracy of the system. The median ARD was 20.27% when the glucose decreased >0.11 mmol/L/min and lower than 10.00% when the glucose changed between 0.06 and 0.11 mmol/L/min, changed <0.06 mmol/L/min, and increased >0.11 mmol/L/min. The sensor glucose was higher than the venous BG when the glucose was decreasing and lower than the venous BG when the glucose was increasing, suggesting that the lead lag phenomenon occurs when there are changes in the glucose concentration [11–13].

The system measured interstitial glucose for up to 14 days using wired enzyme glucose sensing technology, which was first introduced in the FreeStyle Navigator CGM system. The system is factory calibrated, so there was no need for additional calibration during the 14 days. Children and their parents easily accept this system because it is associated with less pain than finger pricks. The study by Edge et al. showed favourable acceptability of the system among children and/or their caregivers [5]. Additionally, no swelling and skin redness associated with the insertion site were reported during the 14 days of use in our study, even for the young child aged 1 year, the youngest to have been reported thus far. The study by Edge et al. reported mild to moderate adverse events related to the device in 6% (5/89) of participants, including allergic reaction, blister, pink/mark scabbing, and abrasion on sensor removal, which resolved at study completion. Mild to moderate adverse events associated with insertion sites, including erythema and pain in 13.6% and 4.1% of participants, respectively, and bleeding, bruising, itching, and edema, were each reported on <3% occasions. We did not detect the above side effects in our study and therefore conclude this system is well tolerated in children.

This is the first study to compare sensor glucose with venous BG in a paediatric population, the results of which have clinical implications for individuals with diabetes and for the clinicians who treat them. The agreement between the sensor glucose and the venous BG is good and remains stable over 14 days of use. The device performs better at higher glucose levels, suggesting that its usefulness is in reducing the HbA1c level, so we recommend its use in paediatric population with higher HbA1c to lower the blood glucose. However, the data of this study suggests that the sensor used in the study does not reliably detect hypoglycaemia; the accuracy of this sensor system is in doubt during emergency/critical glucose levels and does not serve the purpose in the paediatric population, so we should not recommend its use in detecting hypoglycaemia.

## Figures and Tables

**Table 1:** Baseline characteristics of the participants.

	Mean ± SD	Median	Range
Age (years)	8.69 ± 4.01	12.42	0.83~14.58
BMI (kg/m^2^)	15.50 ± 2.80	15.71	11.19~20.7
Insulin dose (IU/kg/d)	1.0 ± 0.3	1.0	0.6~1.3
HbA1c (%) (mmol/mol)	11.62 ± 2.85 (103.50 ± 7.65)	12.10 (108.74)	7.00~17.20 (53.00~164.48)

BMI: body mass index.

**Table 2:** Difference analysis compared with venous reference under various conditions.

	*n*	MARD (%)	*P* value^∗^	CEG	
				A	B
Total	469	11.67	─	94.67	5.33
Days of wear					
2	80	11.53	0.25	93.75	6.25
3	83	9.66		95.18	4.82
6	87	11.79		96.55	3.44
7	88	10.89		96.59	3.41
12	65	13.18		95.38	4.62
13	66	13.92		89.39	10.61
Glucose level					
<4 mmol/L	79	16.89	<0.0001	91.14	8.86
4~10 mmol/L	292	11.58		94.52	5.48
>10 mmol/L	98	7.79		97.96	2.04
Time interval					
7:00~21:00	273	10.88	0.03	95.24	4.76
21:00~7:00	196	12.77		93.88	6.12

^∗^Wilcoxon/Kruskal-Wallis test. MARD: mean absolute relative difference; CEG: consensus error grid.

**Table 3:** The CCC index of sensor blood glucose compared with venous blood glucose.

	*n*	CCC
Overall	469	0.97 (0.96-0.97)^∗^
Glucose level		
<4.0 mmol/L	79	0.49 (0.30-0.64)
4.0~10 mmol/L	292	0.87 (0.84-0.90)
>10 mmol/L	98	0.92 (0.88-0.94)
Days of wear		
2-3	163	0.97 (0.97-0.98)
6-7	175	0.98 (0.97-0.98)
12-13	131	0.94 (0.91-0.95)

CCC: concordance correlation coefficient. ^∗^95% confidence interval.

## Data Availability

The data used to support the findings of this study are available from the corresponding author upon request.

## References

[1] American Diabetes Association (2017). Standards of medical care in diabetes-2017. *Diabetes Care*.

[2] Dovč K., Bratina N., Battelino T. (2015). A new horizon for glucose monitoring. *Hormone Research in Paediatrics*.

[3] Bailey T., Bode B. W., Christiansen M. P., Klaff L. J., Alva S. (2015). The performance and usability of a factory-calibrated flash glucose monitoring system. *Diabetes Technology & Therapeutics*.

[4] Ji L. N., Guo X. H., Guo L. X., Ren Q., Yu N., Zhang J. (2016). A multicenter evaluation of the performance and usability of a novel glucose monitoring system in Chinese adults with diabetes. *Journal of Diabetes Science and Technology*.

[5] Edge J., Acerini C., Campbell F. (2017). An alternative sensor-based method for glucose monitoring in children and young people with diabetes. *Archives of Disease in Childhood*.

[6] Szadkowska A., Gawrecki A., Michalak A., Zozulińska-Ziółkiewicz D., Fendler W., Młynarski W. (2018). Flash glucose measurements in children with type 1 diabetes in real-life settings: to trust or not to trust?. *Diabetes Technology & Therapeutics*.

[7] Deja G., Kłeczek M., Chumięcki M., Strzała-Kłeczek A., Deja R., Jarosz-Chobot P. (2018). The usefulness of the FlashStyle Libre system in glycemic control in children with type 1 diabetes during summer camp. *Pediatric Endocrinology, Diabetes, and Metabolism*.

[8] Cengiz E., Tamborlane W. V. (2009). A tale of two compartments: interstitial versus blood glucose monitoring. *Diabetes Technology & Therapeutics*.

[9] Lodwig V., Heinemann L. (2003). Continuous glucose monitoring with glucose sensors: calibration and assessment criteria. *Diabetes Technology & Therapeutics*.

[10] Rebrin K., Sheppard N. F., Steil G. M. (2010). Use of subcutaneous interstitial fluid glucose to estimate blood glucose: revisiting delay and sensor offset. *Journal of Diabetes Science and Technology*.

[11] Boyne M. S., Silver D. M., Kaplan J., Saudek C. D. (2003). Timing of changes in interstitial and venous blood glucose measured with a continuous subcutaneous glucose sensor. *Diabetes*.

[12] Jansson P. A., Fowelin J., Smith U., Lonnroth P. (1988). Characterization by microdialysis of intracellular glucose level in subcutaneous tissue in humans. *American Journal of Physiology-Endocrinology and Metabolism*.

[13] Sternberg F., Meyerhoff C., Mennel F. J., Mayer H., Bischof F., Pfeiffer E. F. (1996). Does fall in tissue glucose precede fall in blood glucose?. *Diabetologia*.

